# Piggybacking on nature: exploring the multifaceted world of porcine β-defensins

**DOI:** 10.1186/s13567-025-01465-4

**Published:** 2025-03-03

**Authors:** Arthur Nery Finatto, François Meurens, Matheus de Oliveira Costa

**Affiliations:** 1 Large Animal Clinical Sciences, Western College of Veterinary Medicine, University of Saskatchewan, Saskatoon, SK S7N 5E2 Canada; 2 Swine and Poultry Infectious Diseases Research Center, Faculty of Veterinary Medicine, University of Montreal, St. Hyacinthe, QC J2S 2M2 Canada; 3 Department of Veterinary Microbiology and Immunology, Western College of Veterinary Medicine, University of Saskatchewan, Saskatoon, SK S7N 5E2 Canada; 4 Department of Population Health, Faculty of Veterinary Medicine, Utrecht University, Utrecht, The Netherlands

**Keywords:** Antimicrobial resistance, infection control, bactericidal activity, immunomodulatory features, swine, sustainability

## Abstract

Porcine β-defensins (pBDs) are cationic peptides that are classically associated with the innate immune system. These molecules yield both antimicrobial and immunomodulatory properties, as evidenced by various in vitro and animal trials. Researchers have revealed that enhancing pBD expression can be achieved through dietary components and gene editing techniques in pigs and porcine cell models. This state-of-the-art review aims to encapsulate the pivotal findings and progress made in the field of pBD over recent decades, with a specific emphasis on the biological role of pBD in infection control and its usage in clinical trials, thereby offering a new landscape of opportunities for research aimed at identifying prophylactic and therapeutic alternatives for both swine medicine and translational purposes.

## Introduction

Antimicrobial resistance and infection control are global public concerns [1–4]. The effectiveness of antibiotic drugs, which were once widely employed in human and veterinary medicine, has significantly declined over time such that their ability to mitigate microbial infections is no longer as potent as it was observed when they were first introduced in 1928 with the discovery of penicillin [5, 6]. Antibiotic-resistant bacteria are causing concern among healthcare professionals and emotional distress within communities [7, 8]. Hospitalized patients are experiencing infections that no longer respond to existing antibiotics, resulting in untreatable infections and high mortality [8–10]. The interplay between the indiscriminate use of antibiotic drugs and antimicrobial resistance is a cause for concern [1, 3, 4, 11–13], particularly due to the potential for horizontal gene transfer [14, 15]. Horizontal gene transfer allows antibiotic resistance genes to move from one bacterium to another, even between different bacterial species [16, 17]. Such transfer may occur at the farm, wildlife, and urban levels, potentially leading to the acquisition and exchange of resistance genes by human pathogens [16, 18]. The exploration of alternatives to traditional antibiotics, which are less likely to cause resistance, is imperative. The large-scale production of animal protein continues to depend on the use of antibiotic drugs to address animal health issues and promote herd welfare [2, 19]. Furthermore, the environmentally friendly and resource-intensive nature of meat production poses significant challenges for sustainability and food security in the future [20, 21].

**Figure 1:**
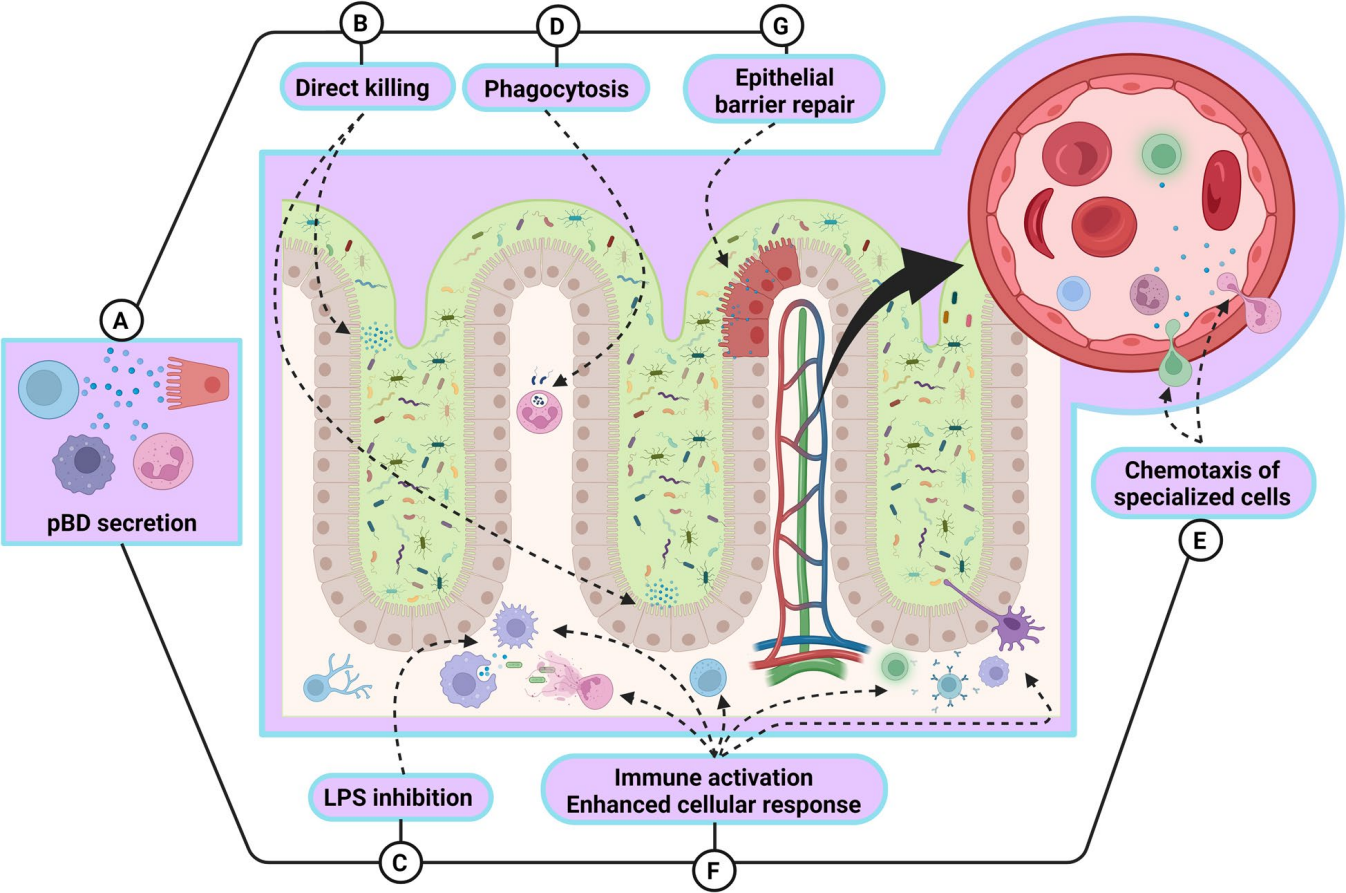
**Multifunctional roles of pBD.** Porcine β-defensins (pBDs) are secreted by a variety of specialized cells (“A”), including B and T lymphocytes, macrophages, neutrophils, and epithelial cells. The roles of pBDs extend beyond direct antimicrobial activity against bacteria, viruses, and fungi (“B”); pBDs also possess the ability to neutralize lipopolysaccharides (LPS) (“C”). These findings suggest that pBDs play a pivotal role in immunomodulating host cells, triggering immune activation and boosting cellular immune responses, thereby enhancing the ability of the host to combat target pathogens. Some of these mechanisms include enhanced phagocytosis (“D”), chemotaxis of specialized cells to sites of infection (“E”), fine-tuned secretion of inflammatory cytokines (“F”), and the strengthening and maintenance of epithelial barrier integrity through the induction of tight junctions (“G”).

Host defence peptides (HDPs) have gained increased attention because of their combined antimicrobial and immunomodulatory properties [22, 23] (Figure 1). HDPs are expressed by the majority of eukaryotic organisms [24], ranging from arthropods to higher mammals [25–30], either in response to microbial stimuli or continuously [31–35], and are secreted at specific sites in the body [36, 37]. These peptides modulate the microbiome and protect the host against infections by inhibiting the growth of microbial cells and orchestrating the host immune response [22, 36, 38–40]. There are few bacterial mechanisms of resistance against these HDPs [41, 42], which demonstrates another advantage over the antibiotics currently used [35]. Biotechnological approaches for the discovery and chemical synthesis of HDPs constitute a new scientific frontier to explore, with the ultimate goal of overcoming the “silent pandemic” of antimicrobial resistance [43].

This literature review focused on porcine β-defensins (pBDs) is timely as it compiles numerous studies into a single review focused on pigs, which are among the most important species for translational health studies, and pork is one of the most consumed animal proteins by humans worldwide [44–47]. Additionally, amid frequent accusations that swine production contributes to the emergence of drug-resistant microbes and is negligent in addressing this issue [15, 48–50], our review highlights the extensive efforts undertaken to address this challenge.

Here, we provide a summary of the structural, biochemical, and biological characteristics of pBDs, as well as recent advances in the use of these peptides to treat and prevent infections. pBDs exhibit swift and wide-ranging antimicrobial capabilities [51], possess immunomodulatory functions [52], show minimal toxicity to host cells [53, 54], maintain stability across diverse environments [55], have a low likelihood of promoting resistance [42], and hold promise for a range of clinical and preventive applications [23, 51, 56–59]. Our aim is to describe the mode of action of β-defensins (BDs), highlight recent progress in this field, and encourage further research. We have structured this review to highlight the innate and acquired immunity mechanisms by which pBDs are involved at both the mucosal and systemic levels, their unique biological properties, in vitro attempts to induce their expression, applications in clinical trials to address infectious diseases, and how new BDs can promote sustainability and help fight antimicrobial resistance.

## Porcine innate immunity and host defence peptides

The porcine innate immune system is similar to that of most mammals and differs in only a few mechanisms or characteristics [46, 60–62]. One example, among others, is the expression of toll-like receptor (TLR) 7 and TLR9, which are restricted to plasmacytoid dendritic cells (pDCs), unlike in mice, where they are also expressed on conventional dendritic cells (cDCs) [60, 63]. Unlike mice [64, 65], pigs have a functional *toll-like receptor 8* (*TLR8*) gene. This receptor can help the immune system of pigs recognize whether pathogens are alive or dead [66]. This ability leads to improved responses from follicular helper T (T_FH_) cells and antibody production when animals are challenged with live bacterial vaccines [66]. Another difference in vaccine development is that, unlike mice and humans, swine lack interleukin (IL)-4, which directs the development of the T_H_2 phenotype [67]. Instead, research has demonstrated that IL-4 inhibits antibody and IL-6 secretion and suppresses the antigen-stimulated proliferation of B cells [67]. In swine, IL-13 affects the role of IL-4 in the development of monocyte-derived dendritic cells (MoDCs) [68]. Pigs have increased diversity of type I interferons, with 39 genes compared with 19 in humans [69, 70]. Additionally, pigs have a duplication of the *IL-1β* gene (*IL-1β* and *IL-1βL*), which does not induce nitric oxide synthase 2 (NOS2) in response to lipopolysaccharides (LPS), as in mice [60, 71, 72]. Pigs also have more cathelicidin genes (11 genes) than humans do, with only one reported gene [70, 73], and thus far, no α-defensins have been identified in swine [60, 74].

Host defence peptides are components of the innate immune system that assume the role of frontline defenders in the early stages of infection. Through direct antimicrobial activity [51], immunomodulation [52], or wound healing [75], HDPs efficiently modulate the commensal microbiome and protect the surrounding host cells [37]. This versatile set of attributes enables the innate immune system to adapt its defence mechanisms precisely to a specific pathogen type or infection stage.

Swine produce a variety of HDPs, including cathelicidin, natural killer (NK)-lysin, hepcidin, liver expressed antimicrobial peptide-2 (LEAP-2), peptidoglycan recognition proteins (PGRPs) and BDs [38]. Cathelicidins typically have an N-terminal signal peptide, a cathelic domain, and a C-terminal antimicrobial domain. They are known for their strong antibacterial activity [76, 77], immunomodulatory properties [78], low haemolytic activity [79], high cytotoxicity towards drug-resistant bacteria [80, 81], and limited antiviral activity against porcine epidemic diarrhoea virus (PEDV) [82]. To date, 11 porcine cathelicidin isoforms have been identified, which are divided into 4 categories on the basis of the biochemical and conformational characteristics of the molecules: PR-39, PG1-5 (protegrins), PF1-2 (prophenins), and porcine myeloid antimicrobial peptides (PMAPs) [77]. All four groups have distinct cellular origins, structural peculiarities, and unique biological features. Shi et al. [77] provided a detailed examination of the characteristics and applications of these peptides in swine. Currently, with advances in molecular engineering techniques, new cathelicidins are being discovered, and modifications of known cathelicidin sequences are being conducted to increase their performance and reduce the risk of microbial resistance [83, 84].

Porcine NK-Lysine presents a linear, amphipathic structure. It has broad-spectrum antimicrobial activity [85–89], has little haemolytic activity against host cells [85], can increase the abundance and distribution of intestinal tight junctions [85], and can modulate the expression of inflammatory cytokines such as IL-6, tumour necrosis factor (TNF)-α, nuclear factor-κB (NF-κB), Caspase 3, and Caspase 9 [85].

Hepcidin contains a conserved disulfide-bonded structure and is cysteine-rich. It is synthesized by liver cells and possesses antimicrobial activity, as shown by in vitro studies [90–92]. Although its primary function is to regulate iron activity [90], the release of hepcidin is an immune strategy to deprive microbes of iron, thus reducing the growth rate of iron-dependent microbes [90]. This iron-linked host defence phenomenon is characterized by an acute decrease in the plasma iron concentration at the onset of inflammatory responses to infection or other proinflammatory stimuli [90]. Initially, Ganz [90] proposed that the mechanism of action of hepcidin involves iron retention in macrophages and a reduction in iron absorption in the intestine.

Liver-expressed antimicrobial peptide 2 (LEAP-2), a typically cationic and amphipathic peptide, was first characterized for its antimicrobial activity [93, 94]. Research has demonstrated that swine infected with *Salmonella enterica* serovar Typhimurium exhibit inducible expression of LEAP-2 in bone marrow and intestinal tissues [94]. Recent research has indicated that these peptides may act as endogenous blockers of growth hormone secretagogue receptor 1a (GHS-R1a), acting both as endogenous competitive antagonists of ghrelin and inverse agonists of constitutive GHS-R1a activity [93]. This dual activity affects food intake and hormonal secretion, which makes LEAP-2 a promising target for the development of obesity drugs [93]. Thus, like other HDPs, LEAP-2 has a dual biological role.

Peptidoglycan recognition proteins (PGRPs) are produced by many species of animals, including swine [38, 95–98]. These proteins are involved in the activation of toll-like receptors (TLRs) [97, 99], the induction of proteolytic cascades with the goal of producing antimicrobial products (e.g., β-defensins) [95], and direct bactericidal activity [100]. Sang et al. [95] reported that porcine PGRP-L1 and PGRP-L2 play important roles in the onset of antimicrobial peptide β-defensin-1 (pBD-1) expression under experimental conditions. These findings demonstrated that the silencing of the *pPGRP* gene downregulates pBD-1 [95]. In addition to revealing the intracellular pathways involved in the production of pBD-1, these findings shed light on the complexity and interdependence of the pathways involved in HDP production.

Currently, 28 pBDs have been described on swine chromosomes 7, 14, 15 and 17 [74] (Table 1). These molecules are cysteine-rich peptides that are less than 100 amino acids long and contain disulfide bridges and common end-to-end macrocyclization in their structures [38]. These biochemical properties confer hyperstability at high temperatures and salt concentrations, the presence of serum and the degradation of proteases [55]. There is evidence that the genetic codons responsible for encoding BDs in swine exhibit the highest level of sequence conservation compared with codons encoding other host defence peptides [38, 101, 102]. This finding is consistent with the hypothesis that the production of BDs has offered adaptive benefits throughout evolution and may have contributed to the persistence of these genetic traits to the present day [103, 104].

**Table 1:** **Genomic locations of porcine β-defensin (pBD) genes in the pig genome (*Sscrofa 11.1*)**

pBD	Gene symbol	Chr	Location
pBD-1	DEFB1	15	NC_010457.5 (38076057..38077899)
pBD-2	Unknown	Unknown	Unknown
pBD-110	DEFB110	7	NC_010449.5 (44019705..44024322, complement)
pBD-112	DEFB112	7	NC_010449.5 (44031495..44041549, complement)
pBD-113	DEFB113	7	NC_010449.5 (43999119..44000402, complement)
pBD-114	DEFB114	7	NC_010449.5 (43982219..43993156, complement)
pBD-115	DEFB115	17	NC_010459.5 (35029031..35034148)
pBD-116	DEFB116	17	NC_010459.5 (35063977..35067003, complement)
pBD-121	DEFB121	17	NC_010459.5 (35154177..35155772, complement)
pBD-123	DEFB123	17	NC_010459.5 (35185257..35190686)
pBD-124	DEFB124	17	NC_010459.5 (35200560..35205507, complement)
pBD-125	DEFB125	17	NC_010459.5 (35003914..35010677, complement)
pBD-127	DEF127	17	NC_010459.5 (34973682..34975738, complement)
pBD-128	DEFB128	17	NC_010459.5 (34948185..34965455)
pBD-129	DEFB129	17	NC_010459.5 (34939180..34942357, complement)
pBD-133	DEFB133	7	NC_010449.5 (43970402..43975033, complement)
pBD-134	DEFB134	14	NC_010456.5 (15171675..15176787, complement)
pBD-135	DEFB135	14	NC_010456.5 (15143118..15145096)
pBD-136	DEFB136	14	NC_010456.5 (15109961..15110778, complement)
pBD-119	LOC100157086	17	NC_010459.5 (35131724..35142093, complement)
pBD-104A-like	LOC106504227	15	NC_010457.5 (37960250..38006690)
pBD-105-like	LOC106506206	15	NC_010457.5 (37982133..37989094, complement)
pBD-108B-like	LOC110256909	15	NC_010457.5 (38135983..38150772)
pBD-130-like	LOC110257049	15	NC_010457.5 (38189899..38195975)
pBD-15-like	LOC110257188	15	NC_010457.5 (37989086..37996091)
pBD-110-like	LOC110261742	7	NC_010449.5 (44008804..44009329, complement)
prepro-pBD-3	LOC404703	15	NC_010457.5 (37960248..38065632)
prepro-pBD-108-like	LOC692190	17	NC_010459.5 (35043632..35044109, complement)

This table provides detailed genomic information on pBD genes, including their gene symbols, chromosome numbers, and specific chromosomal locations within the pig genome assembly Sscrofa 11.1. The prefix “*prepro*” denotes that the protein is synthesized as a precursor molecule requiring processing for activation. The suffix “*like*” indicates that the defensin’s sequence or structure resembles another defensin, on the basis of NCBI annotations. The specific evolutionary relationships of “*like*” genes (e.g., paralogue or orthologue status) could not be determined from the available data.

β-defensins are produced by epithelial cells on mucosal surfaces and by granule-containing leukocytes, including neutrophils, natural killer (NK) cells, and cytotoxic T lymphocytes [36]. There is increasing evidence that different cell types express different pBD genes [105], each expressing pBDs at different levels [37, 105]. Veldhuizen et al. [37] reported that pBD-1 gene expression increases from the proximal to the distal part of the small intestine. This may constitute a strategy to modulate the microbiome topographically, providing specific homeostasis for each mucosal niche [37].

The regulation of pBD expression is believed to involve a complex interplay of numerous factors, including cytokine signalling [106–108], hormonal mediation [109], dietary factors [110–112], and the fine-tuning of gene expression through epigenetic modifications [113]. To date, most of the knowledge concerning the regulation of pBD expression comes from in vitro or comparative studies involving animal species other than swine.

### Antimicrobial role of β-defensins

#### Antimicrobial spectrum

pBDs have broad-spectrum antimicrobial activity, inhibiting bacterial, viral and fungal growth. The inhibition of both Gram-positive and Gram-negative bacteria has been described [51]. In bacteria, the inhibitory capacity of pBDs may be influenced by the presence or absence of a cell wall, as well as by the presence, absence, and amount of lipopolysaccharide and peptidoglycan layers. One example of this is the first discovered pBD, pBD-1. It more efficiently inhibits the growth of Gram-negative bacteria than Gram-positive, as evidenced by in vitro trials [38, 114]. pBD-2, on the other hand, has broad activity against both targets [51]. Assays involving the inhibition of viral growth show modest activity against viral replication [51]. Notably, pBD-3 suppressed between 50 and 80% of porcine reproductive and respiratory syndrome virus (PRRSV) infections in an in vitro study involving porcine lung cells [115]. Intriguingly, pBD-1 and pBD-2 do not suppress viral activity at the same rate as pBD-3 does [115]. These findings demonstrate how the chemical structure of pBDs significantly impacts their activity spectrum. Finally, pBD-1 was the only molecule shown to have fungicidal activity against *Candida albicans* [114].

#### Antimicrobial mechanisms

**Figure 2:**
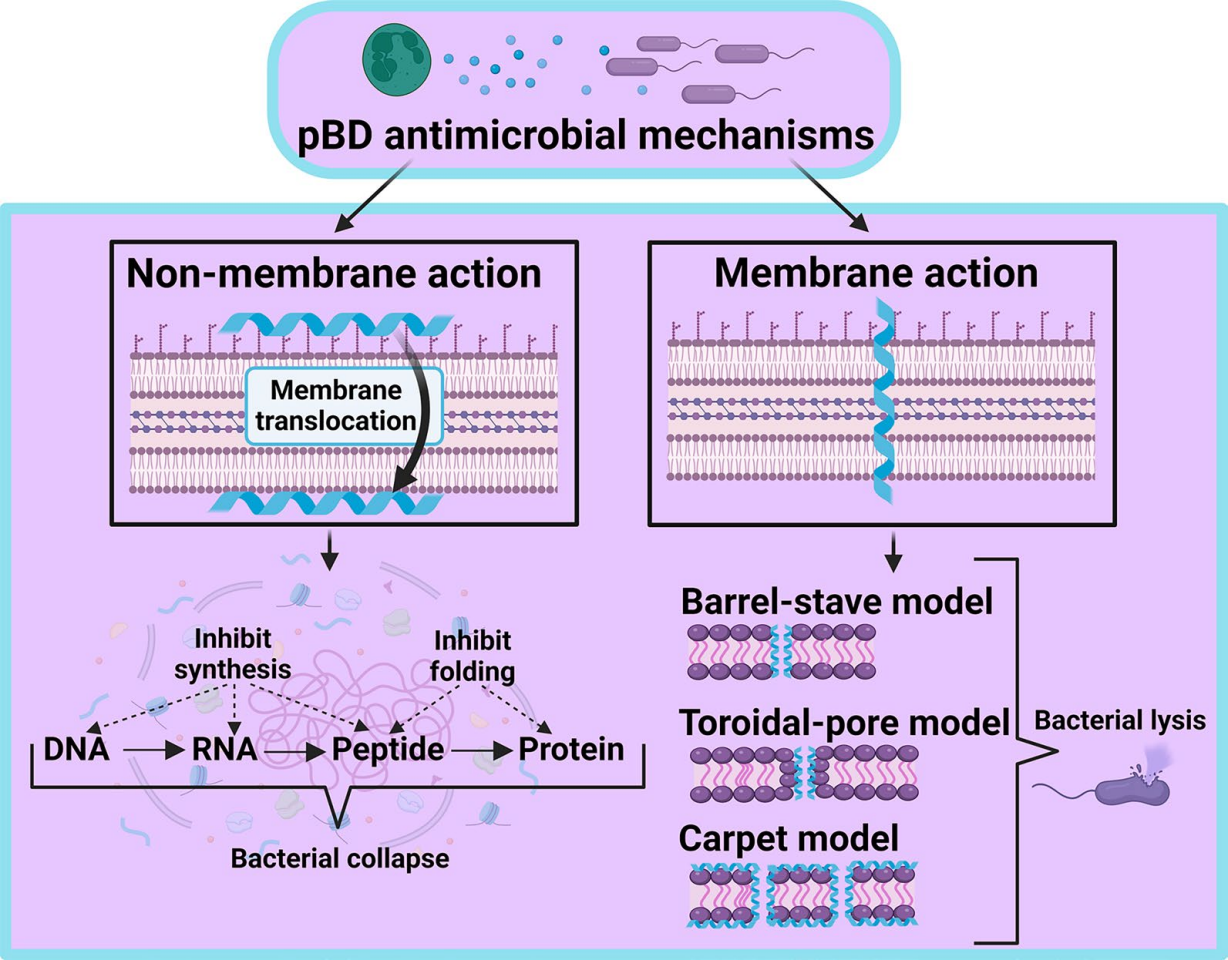
**Antimicrobial mechanisms of pBD.** This figure illustrates the membrane and non-membrane action mechanisms of porcine β-defensins (pBDs) in the inhibition of microbial growth. The barrel-stave model shows that pBDs form barrel-like channels in the bacterial membrane. The toroidal pore model depicts pBDs connecting the outer and inner lipid layers, disrupting the membrane structure. The carpet model demonstrates that pBDs coat the bacterial surface, disrupting the bilayer structure. The non-membrane action model illustrates that pBDs impair DNA transcription, translation, and protein folding through intracellular interactions.

Although the majority of the knowledge regarding antimicrobial mechanisms involving antimicrobial peptides (AMPs) is derived from human molecules, it is worth considering these mechanisms in swine, given the considerable similarities between these two species [46, 69]. Currently, three membrane disruption mechanisms have been proposed, namely, the barrel-stave, toroidal, and carpet models [116–120] (Figure 2). The barrel-stave pore model postulates that defence peptides assemble into dimers or multimers after binding to the negatively charged bacterial membrane [121]. This peptide array then traverses the cell membrane and reaches the interior of the bacterial cell. The assembly of peptides is a critical step in the formation of pores, and assembled peptides create barrel-like channels reminiscent of staves. In the toroidal pore model, peptides create a monolayer that bridges the outer and inner lipid layers of the membrane [122]. Conversely, the carpet model explores the possibility that defence peptides may act as surfactants, covering the surface of the bacterial cell and disrupting the membrane bilayer [123]. Additionally, the existence of a threshold concentration for optimal efficiency is suggested.

In addition to the previously mentioned mechanisms, recent research involving electron microscopy and gene expression analysis revealed that *E. coli* membranes were broken, holed, and wrinkled after treatment with pBD-2 [124]. This study also suggested that pBD-2 interferes with *E.* coli DNA transcription and translation [124] (Figure 2).

## Immunomodulatory features of β-defensins

Immunomodulatory features have also been extensively associated with BDs across many species. These peptides play a role in reducing exacerbated inflammatory responses [125, 126], wound healing [127], chemokine production and chemotaxis of specialized cells [128, 129], and interactions with effector cells of the innate and adaptive immune systems [39, 125, 126, 128, 129].

Transgenic founder pBD-2-expressing mice are relatively resilient to *Salmonella enterica* subsp. *enterica* serovar Typhimurium infection as a consequence of the direct bactericidal effect of pBD-2 [23]. Compared with control mice, animals that expressed pBD-2 presented with reduced epithelial damage, intestinal submucosal oedema and less neutrophil accumulation in the caecum (unable to express pBD-2). Systemic effects of pBD-2 in mice include reduced levels of circulating IL-6, TNF-α, IL-1β, and IL-12 following inoculation with *S.* Typhimurium or LPS [23]. Genetically edited mouse macrophages that produce pBD-2 suppress cytokine expression by inhibiting the LPS-activated NF-κB signalling pathway [23]. Zhang et al. [130] used IPEC-J2 cells to demonstrate that pBD-2 reduces cell apoptosis; downregulates the expression of the apoptosis-related genes *Cox-2* and *Caspase-3*; decreases the expression of *IL-6*, *IL-8*, *IL-1α*, and *C-X-C motif chemokine ligand 2* (*CXCL2*) and *C–C motif chemokine ligand 20* (*CCL20*); reduces the expression of *transforming growth factor-β-activated kinase 1* (*TAK-1*); and inhibits *NF-κB p65*. The authors suggested that alleviation of inflammation involves cytoplasmic interference with the TAK1-NF-κB signalling pathway. Moreover, the findings of Zhang et al. [130] corroborate Huang et al. [23], suggesting that pBD-2 inhibits the NF-κB inflammatory pathway.

To address the intracellular mechanisms involving pBD-2 and the mitigation of inflammation, Huang et al. [23] conducted a study using fluorescently labelled pBD-2. The authors demonstrated that pBD-2 inhibits *vasohibin-1* (*VASH1*), a known angiogenesis inhibitor [131]. They also demonstrated how LPS could induce inflammation through the AKT/NF-κB signalling pathway, while pBD-2 could disrupt this mechanism, suppressing the inflammatory response.

Su et al. [132] have provided further evidence of the critical role played by the NF-κB signalling pathway in the biological function of pBD-114. pBD-114 activation through the TLR-activated NF-κB signalling pathway suppressed inflammation and reduced epithelial cell apoptosis in both mice and IPEC-J2 cells following challenge with enterotoxigenic *E. coli* K88 CVCC 225 [132].

A study in mice demonstrated the potential prophylactic effects of murine β-defensin 2 (mBD-2) during *Mycobacterium tuberculosis* infection [58]. Researchers have reported that mBD-2 functions as a liaison between the innate and adaptive immune systems, inducing a Th_1_ response through the upregulation of interferon γ (IFN-γ), IL-12, and IL-6 in dendritic cells [58]. Another study explored gene therapy with a recombinant adenovirus vector to induce the overexpression of human β-defensin 3 (hBD-3), human cathelicidin, and mouse TNF-α. This strategy was used for the prevention of experimental latent tuberculosis reactivation in a murine model [133]. Gene therapy with hBD-3 reduces pneumonia, possibly through increased levels of IFN-γ and inducible nitric oxide synthase (iNOS) expression and the chemoattraction of lymphocytes and macrophages [133]. The role of pBDs in the chemotaxis of leukocytes, activation of immune cells, and regulation of adaptive immunity in swine has remained underexplored. This gap has the potential to reveal new strategies for prophylaxis and treatment (e.g., enhancing the antibody response to vaccines) [36, 58, 134].

## Attempts to induce pBD expression in porcine epithelial cells

Zeng et al. [112] demonstrated that the expression of pBD-2 and pBD-3 mRNAs can be increased through exposure of IPEC-J2 cells, 3D4/31 macrophages and primary monocytes to butyrate. Similarly, butyrate analogues such as glyceryl tributyrate, benzyl butyrate, and 4-phenylbutyrate could also induce the expression of pBD-2 and pBD-3. The expression of pBD-1 remained largely unaltered in IPEC-J2 cells, 3D4/31 macrophages and primary monocytes.

Using IPEC-J2 cells, Mao et al. [111] measured the mRNA expression of pBD-1, -2 and -3 after 12 h of exposure to either l-isoleucine or zinc sulphate. The expression of all pBDs increased following treatment. Interestingly, high doses (≥ 250 µg/mL of l-isoleucine or ≥ 250 µmol/mL of zinc sulphate) resulted in an abrupt decrease in gene expression for the three target pBD genes. These findings suggest that the increase in pBD expression is not dose dependent. This statement is especially pertinent in swine medicine, as zinc oxide supplementation is a widespread practice used to control postweaning diarrhoea (PWD) [135]. While not yet fully explored and validated in vivo [111], this insight may have practical applications in the field. Wang et al. [110] demonstrated that IPEC-J2 cell expression of pBD-1, pBD-2 and pBD-3 is dose- and time-dependent following exposure to L-threonine at concentrations up to 1 mM. At this concentration, the expression levels of the pBDs were reduced. The same study also revealed that pBD expression inversely correlated with the gene expression of *IL-6*, *IL-8*, *IL-1β* and *TNF-α*, emphasizing the regulatory role of pBD.

Xue et al. [136] reported that recombinant mature porcine IL-22 (mpIL-22) inhibited the infection of porcine epithelial cells with porcine rotavirus (PorRV) and enteric coronaviruses (PEDV and transmissible gastroenteritis virus (TGEV)) through the overexpression of pBD-2, IL-18 and IFN-λ. According to the authors, the mechanism of pBD-2 overexpression was mediated by STAT3, as suggested by the phosphorylation of *signal transducer and activator of transcription 3* (STAT3) at phosphorylation sites Ser727 and Tyr705 in IPEC-J2 cells. To verify this outcome, the authors further used S3I-201, a STAT3-specific inhibitor, to verify how mpIL-22 behaves in the absence of STAT3. By doing so, they confirmed that the presence of S3I-201 can abolish the antiviral activity of mpIL-22, inhibiting the production of pBD-2 and promoting viral growth. These findings shed light on the transcription mechanism of pBD-2. DNA phosphorylation is possibly involved in the regulation of pBD-2 expression, affecting the accessibility of the DNA for the transcriptional machinery to bind and initiate transcription.

## In vivo advances in the use of pBD overexpression to mitigate disease progression

One of the first clinical trials shedding light on the role of pBD-1 as a potential disease mitigator in pigs was conducted by Elahi et al. [59]. The authors reported that pBD-1 expression was significantly associated with protection against *Bordetella pertussis* in 4- to 5-week-old pigs but not in newborns, independent of whether they received colostrum. The authors validated their findings in vitro, verifying that pBD-1 could inhibit the growth of *B. pertussis*.

Tang et al. [137] exposed 30 animals to the enterotoxigenic *E. coli* strain 0149:K88 (ETEC) and reported that pBD-2-treated animals presented growth performance comparable to that of neomycin sulphate-treated animals. pBD-2 treatment also induced similar histological, immune, and intestinal microbiome characteristics to those of the antibiotic-treated group.

Huang et al. [57] conducted a clinical trial in which they exposed genetically edited pigs that overexpressed pBD-2 and conventional animals to *Glaesserella* (*Haemophilus*) *parasuis*. Compared with control pigs, pBD-2 pigs presented significantly milder clinical signs and less severe gross pathological changes. Notably, the necroscopic and histological findings were accompanied by reduced loads of *G. parasuis* in tissues, which corroborates the hypothesis that pBD-2 may have mitigated the clinical signs associated with Glässer’s disease [57].

Huang et al. [56] verified that pigs overexpressing pBD-2 had alleviated influenza-A virus-associated clinical signs. In vitro models confirmed that pBD-2 penetrates epithelial cells to interact with the mitochondrial solute carrier family 25 member 4 (SLC25A4) through hydrogen bonding, alleviating cell apoptosis induced by the virus, and that pBD-2 interferes with virus adsorption and post-entry stages in the cell. Although more studies are needed to clarify the adsorption mechanisms by which pBD-2 is involved, overexpressing pBD-2 in pigs led to reduced shedding and transmission between pigs, which may represent an applicable solution for pig farmers.

Yang et al. [138] reported that overexpressing pBD-2 in pigs has an effect on the organ distribution of *Actinobacillus pleuropneumoniae* in a cohabitation clinical trial. They demonstrated that, compared with normal-producing pBD-2 pigs, pBD-2-overexpressing pigs presented reduced bacterial loads in the lungs, although no differences were observed in terms of survival rate. The authors reported that the control pigs had severe congestion, haemorrhages, and oedema in the lungs, whereas only mild congestion and oedema were observed in those of pBD-2-overexpressing pigs.

## Future perspectives

The possibilities surrounding pBDs are vast and largely untapped. Various pBDs have been identified [74], their bactericidal properties against different microorganisms have been established [23, 53, 57, 59, 137], and their potential for immunomodulation has been noted [23, 110–112, 136, 139]. While these advancements are significant for the development of applicable solutions, there is still much to be understood to fully harness the potential of these innate host peptides. Their immunomodulatory capacity remains modestly explored but holds immense long-term benefits for the livestock industry.

To the best of our knowledge, the use of pBDs in combination with a vaccination protocol has not yet been employed in swine. By incorporating a diet that enhances the expression of pBDs and closely monitoring cell dynamics, cytokines, and vaccine antibody responses, researchers can take a major step forward in understanding the role of innate peptides in disease prevention. Such a clinical trial could have far-reaching implications, not only by offering valuable insights into the optimization of existing vaccines in the swine industry but also by providing a potential solution for the prophylaxis of diseases that require specific cellular responses but are not functional via currently available strategies.

The potential for pigs genetically modified to produce pBDs to contribute to scientific breakthroughs is undoubtedly appealing [23, 56, 57, 138]. However, it remains uncertain whether this technology will be embraced by the pork industry [140–142]. While producers and packagers may be inclined to use those animals, consumer acceptance of these products is not always as expected. The market and legislation for genetically modified crops have already been established, but the challenges faced by the market for genetically edited animals appear to be much greater. A survey conducted by the Pew Research Centre revealed that while 50% of Americans said they would be willing to eat genetically modified foods, only 29% said they would be willing to eat meat from genetically edited animals [143]. This attitude is even more pronounced among European citizens, where it was shown that meat consumers support technologies that improve animal welfare standards but dislike excessive manipulation and lack the naturalness of their meat products, such as those represented by gene-editing technologies [144, 145].

The study of editing pBD sequences for chemical synthesis appears to yield promising results and is likely to receive increased attention in the near future [130]. In our opinion, the biochemical engineering of biologically derived molecules presents vast opportunities for enhancing their biological potential in controlling bacterial growth and offers intriguing prospects for reducing the likelihood of developing antimicrobial resistance [146–149].

A critical aspect that must be considered when discussing genetic selection and breeding, gene editing, or simply the administration of pBDs to pigs is the risk of selecting pathogens resistant to pBDs produced by the animals themselves. Although current evidence suggests that pathogens rarely develop resistance mechanisms to pBD in their wild form because of their multiple modes of action [124], this assumption may be overly optimistic. Vigilance is essential, as the potential for resistance development cannot be dismissed [150–152]. Gene editing and selection programs for pigs with high pBD production or clinical administration of synthetic pBDs must rigorously assess the potential risks of fostering antimicrobial resistance, balancing these risks with any health or production benefits. Pathogens may eventually develop resistance to pBDs [41, 42, 59], but we hypothesize that modulating innate and acquired immunity through these compounds as a prophylactic measure is much more difficult to circumvent and represents the most promising area to explore.

Finally, it is crucial to identify the actors who drive efforts to transform these ideas into applicable products for pig farmers and ultimately benefit society as a whole. The development of pBDs requires the collaboration of scientists from various disciplines, including biology, veterinary science, chemistry, and biotechnology. Additionally, legal support, such as patent protection and copyright defense, should be prioritized. To simplify the complex process and streamline marketing strategies, academic research centers should consider partnering with the industry. Properly funded and coordinated efforts to produce pBDs have the potential to increase sustainability in the pork industry and contribute to a more prosperous world.

## Conclusions

Over the past two decades, interest in pBDs has increased due to their potent antimicrobial properties. The results of research in this area have been highly encouraging, demonstrating the ability of pBDs to mitigate numerous production-limiting diseases in the swine industry. This has far-reaching consequences, not only in terms of animal welfare but also in terms of the economic viability and profitability of the productive sector. Moreover, there is abundant evidence to support the immunomodulatory role of pBDs, albeit with less emphasis placed on this aspect than on their antimicrobial activity. Nonetheless, our hypothesis is that the field of immunomodulation holds enormous potential for the development of high-value, applicable technologies.

## Data Availability

Data sharing does not apply to this article as no new data were created or analyzed in this study.
